# Reactive macrophage activation syndrome in a patient with parvovirus B19 infection, lymphocytic lichenoid vasculitis, urticaria and angioedema

**DOI:** 10.4103/0256-4947.59371

**Published:** 2010

**Authors:** Dragica Soldo-Juresa, Maja Radman, Vlatko Pejsa, Velimir Bozikov

**Affiliations:** a From the University Hospital Vuk Vrhovac, Zagreb, Croatia; b From the Department of Endocrinology, University Hospital Centre Split, Split, Croatia; c From the Department of Internal Medicine, University Hospital Dubrava, Zagreb, Croatia

**Figure 1:**
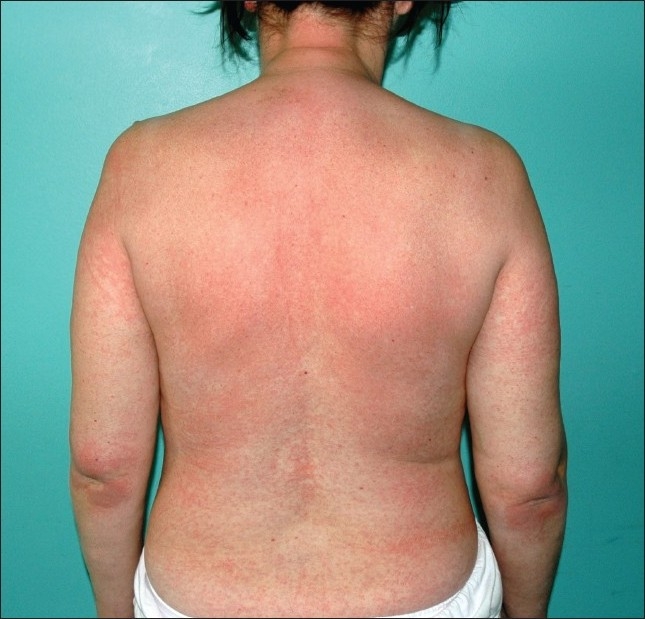
Maculopapular rash.

**Figure 2:**
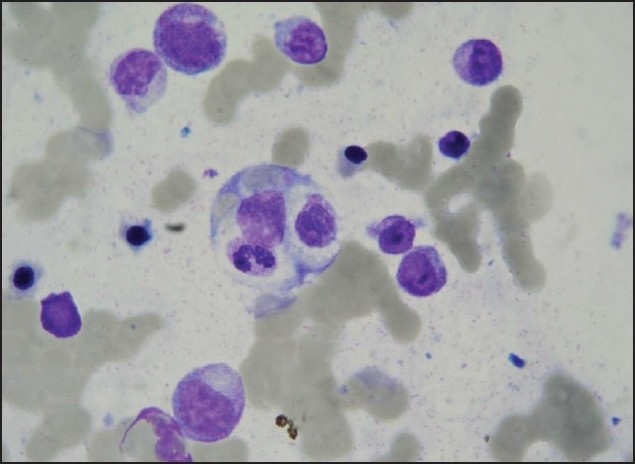
Bone marrow aspirate shows increased number of histiocytes with active hemophagocytosis (Hematoxylin stain ×200 magnification).

**Figure 3:**
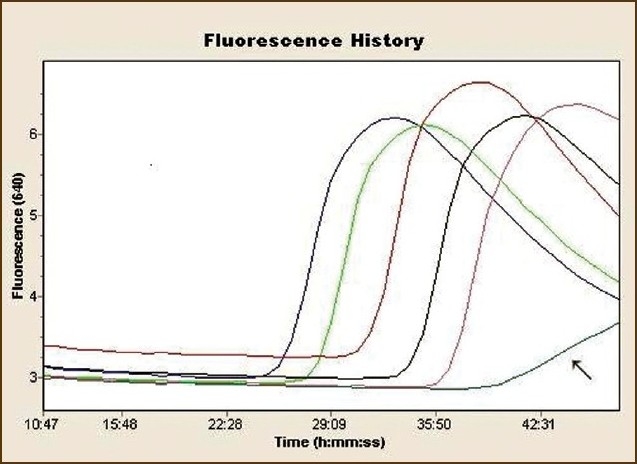
Real-time PCR that confirmed parvovirus B19 (arrow shows negative control sample).

A 47-year-old woman had a history of episodic acute intermittent angioedema and urticaria with moderate pruritus for one month. She was on 10 mg of loratadine daily. She had a fever of 39°C, arthralgia, fatigue, and angioedema of the upper respiratory tract. Laboratory results are shown in Table 1. She had elevated IgE (206 kU/L). The immunoassay for C1-esterase inhibitor was normal. She was treated with H1- and H2-blocking antihistamines, and methylprednisolone intravenously (1 mg/kg/day). Culture results and viral titers were negative except for a high positive titer of specific IgG antibody to parvovirus B19 of 11.1 (positive titer >1). On the third week of hospitalization, she deteriorated rapidly and developed a macular rash on the trunk and extremities with generalised lymphadenpathy, liver dysfunction and disseminated intravascular coagulopathy (DIC) (Figure 1). A skin biopsy specimen was compatible with lymphocytic lichenoid vasculitis. An inflammatory pattern centered on the basal layer of the epidermis and upper dermis in a dense band-like distribution. Direct immunofluorescence showed no IgG, IgA, IgM, C3, C1q and fibrinogen deposits. A bone marrow aspirate showed hemophagocytosis (Figure 2). Parvovirus B19 DNA was detected by the polymerase chain reaction (PCR) in bone marrow (Figure 3). Macrophage activation syndrome was confirmed. The patient was treated with methylprednisolone 250 mg/day intravenously and intravenous immunoglobulin (IVIG) 0.55 g/kg BW/day for five consecutive days, followed by methylprednisolone 1 mg/kg daily. Fresh frozen plasma and enoxaparin were administered. Two days after treatment, she improved. Monthly infusions of IVIG were continued for 6 months. Corticosteroids were tapered gradually to 8 mg of methylprednisolone daily. On periodic follow-up, the patient was quite well without episodes of angioedema and no new skin lesions were seen. Our patient met all criteria for reactive macrophage activation syndrome (rMAS) outlined by Imashuku.^1^

**Table 1:** Laboratory test results.

Laboratory data (normal values in parenthesis)	Admission	Before IVIG^a^	After first IVIG^a^
Erythrocyte sedimentation rate (2-13 mm/h)	57	16	122
White blood cell count (4-10×10^9^/L)	11.8	5.8	11.0
Hemoglobin (120-160 g/L)	132	78	101
Platelet count (140-440×10^9^/L)	203	33	249
C reactive protein (0-5 mg/L)	143	88.2	28.7
Aspartate aminotransferase (7-49 IU/L)	28	720	54
Alanine aminotransferase (7-49 IU/L)	35	500	75
Lactate dehydrogenase (170-430 IU/L)	361	3206	335
Ferritin (10-300 mg/L)	12200	43700	8300
Fibrinogen (2-4.5 g/L)	6.1	1.4	4.0
D-dimer (0.06-0.25 mg/ml)	0.15	4.36	0.30

a IVIG = intravenous immunoglobulin

Acute parvovirus B19 infection can be diagnosed by demonstrating a four-fold rise in serum B19-specific IgG antibody titers, as in our case. B19 specific DNA or antigens can be detected for months or even years after infection.^2^,^3^ We speculate that the presence of acute parvovirus B19 infection was a trigger for rMAS.^4^ Lymphocytic vasculitis is a reactive process.^5^,^6^ Purpura pigmentosa chronica, fixed drug eruption, urticarial vasculitis, allergic vasculitis, and the vasculitis of Sjogren syndrome are known to have lymphocytic vasculitis. We conclude that rMAS might represent a subgroup of patients with systemic inflammatory response amenable to IVIG treatment. Given early, IVIG may interrupt the processes that lead to macrophage overactivation.
